# The Use of First‐Void Urine to Screen Women Aged 60–79 for HPV in the UK: The Catch‐Up Screen Study

**DOI:** 10.1111/1471-0528.70115

**Published:** 2025-12-15

**Authors:** Christine Rake, Emma J. Davidson, Alex Young, Annelie Maskell, Jennifer C. Davies, Marina Flynn, Sofia Vidali, Hannah Mohy‐Eldin, Jiexin Cao, Belinda Nedjai, Una Macleod, Julian Peto, Clare Gilham

**Affiliations:** ^1^ Faculty of Epidemiology and Population Health London School of Hygiene & Tropical Medicine London UK; ^2^ Gynaecological Oncology Research Group, Division of Cancer Sciences, Faculty of Biology, Medicine and Health University of Manchester Manchester UK; ^3^ Department of Obstetrics and Gynaecology St Mary's Hospital, Manchester University NHS Foundation Trust, Manchester Academic Health Science Centre Manchester UK; ^4^ Centre for Reproductive Health University of Edinburgh Edinburgh UK; ^5^ Hull‐York Medical School University of Hull Hull UK; ^6^ Hull and East Yorkshire Hospitals NHS Trust Hull UK; ^7^ Wolfson Institute of Population Health Queen Mary University of London London UK

**Keywords:** catch‐up, cervical screening, first‐void urine, HPV, older women, self‐sampling

## Abstract

**Objective:**

Almost half the deaths from cervical cancer in the UK are among women aged over 65 who were already above the upper age of screening when primary HPV screening was introduced in the UK in 2019. Our aim is to test the feasibility of a national catch‐up HPV testing programme.

**Design:**

This first phase of the Catch‐Up Screen study involved randomizing over 3000 invited participants to receive a urine HPV test and a follow‐up telephone call or text message.

**Setting:**

GP practices in Hull and Manchester, UK.

**Population:**

Women aged 60–79 who have not undergone primary HPV screening.

**Methods:**

Eligible women were selected from GP practice records, and 3074 were invited to provide an at‐home first‐void urine sample for HPV testing.

**Main Outcome Measures:**

Uptake of at‐home urine screening according to screening history, area‐level index of deprivation, and randomised follow‐up method.

**Results:**

Overall, 59% (1816) of invited women returned a urine sample for HPV testing. Response varied by screening history and index of area‐level deprivation, but 39% of those who declined their last invited NHS screen responded favorably and took part in Catch‐Up Screen. Telephone reminders yielded a 5% absolute increase in response compared to the text message arm (*p* = 0.007).

**Conclusions:**

An at‐home first‐void urine sample is a viable method for a national catch‐up HPV test and has the potential to address decreasing national coverage among older women being invited for their last screen.

## 1 Introduction

Testing for high‐risk human papillomavirus (HPV) is the most effective way to identify women at risk of developing cervical cancer [1]. Since 2019, the NHS Cervical Screening Programme (CSP) has offered primary HPV testing to women aged 25–64 years [2]. Women who have a negative HPV test before they stop screening are at much lower risk of developing cervical cancer than those screened by and negative for cytology [3]. There are around 400 cervical cancer deaths every year in the UK among those aged 65 and over [4], most of whom have never been offered an HPV test because they were already above the upper age of screening when primary HPV testing was introduced. In England and Wales, an estimated 1 in 1200 women who had been regularly screened with cytology developed cervical cancer at age 65–84 [5]. Offering a catch‐up HPV test therefore has the potential to prevent future cervical cancer in these older birth cohorts [6].

The Australian cervical screening programme offers an exit test at age 70–74 [7], detecting around 0.8 cervical cancers per 1000 women screened since 2018 [8]. In 2017, a catch‐up clinician‐taken test was offered to all women aged over 70 in Denmark, detecting cervical cancer in 0.5 per 1000 women screened. The unrestricted upper age limit proved controversial, though 94% of those screened were aged under 85 [9]. From early 2025, a vaginal self‐taken swab has been offered to women born between 1947 and 1952 in Stockholm, Sweden. We aim to test the feasibility of offering a catch‐up urine HPV test to English women aged 60–79 who never had a primary HPV test.

Older women face similar barriers to cervical screening as younger women, such as inconvenience and embarrassment, but additionally report discomfort and/or pain due to the speculum examination secondary to post‐menopausal reduced oestrogen levels [10, 11]. To address these barriers, Catch‐up Screen involves women providing a urine sample from the privacy of their home. HPV‐tested first‐void urine samples have been shown to have comparable performance to clinician‐taken cervical samples for the detection of CIN2+ [12, 13, 14]. It is hoped that urine sampling will encourage women to take part who were not screened regularly and who are likely to be at higher risk [5, 15]. The aim is to screen 10 000 women over 3 years. We report on the first year of the project where just over 3000 women were invited to test the study methodology and assess the impact of a telephone vs. text message reminder.

## 2 Methods

### 2.1 Study Population and Invitation

**FIGURE 1:**
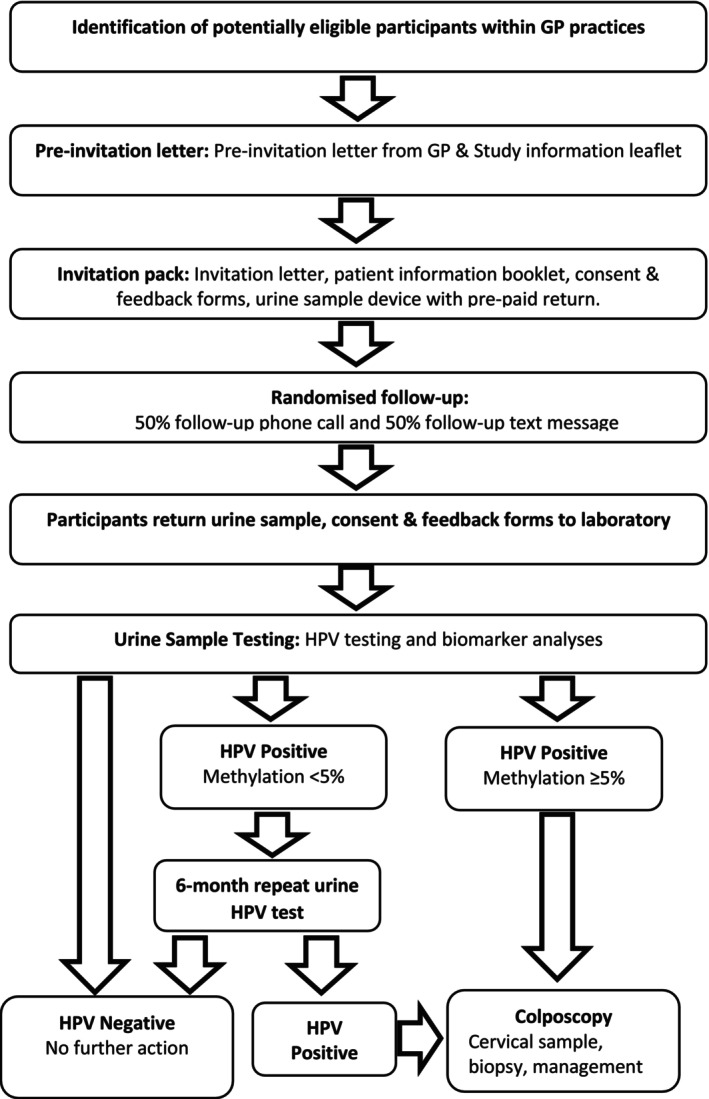
Study flow diagram.

During this first phase of the project, 3074 women were invited to take part between January and December 2024. Figure 1 shows the study flow diagram. Potential participants were selected from records in three GP practices in Hull and Manchester, UK. Women were eligible if they were aged 65–79 and had their last screening invitation before the introduction of primary HPV testing in 2019, or aged 60–64 and did not attend for their last and only invited primary HPV screening test (since 2019). Further eligibility screening excluded those who had undergone a full hysterectomy, dissented from their records being used for research, were unable to give informed consent (including living with dementia), and those living in nursing homes or receiving end‐of‐life care.

Potential participants were first sent a pre‐invitation letter from their GP with an introductory leaflet giving information about HPV and HPV screening. A week or two later, they were sent a full invitation pack containing a letter from their GP, a full patient information booklet, a consent form, a first‐void urine collection device (10 mL Colli‐Pee®, DNA Genotek Inc.), a feedback form, a pre‐paid return box, and a thank you postcard from Yorkshire Cancer Research. The Colli‐Pee device allows first‐void collection of urine directly into a tube for immediate preservative mixing without stopping the flow of urine.

During this first year of the project, the participants were randomised to receive either a text message or a phone call from their GP practice within a few days of receipt to give them the opportunity to discuss the study and ask questions. Up to 3 telephone calls were attempted for each participant in the telephone reminder arm. To ensure everyone received an opportunity to ask questions, some weeks after all patients had been invited from the practice, a final text message was sent to those who had not responded. Those who had no mobile phone number recorded on their notes were called by landline telephone.

### 2.2 HPV Testing

Participants returned their urine sample, consent form, and feedback form by pre‐paid post to the Molecular Epidemiology Laboratory at Queen Mary University London. The BD Onclarity HPV assay has positivity cycle thresholds of ≤ 38.3 for HPV16 and ≤ 34.2 for all other genotypes and the β‐globin internal control which are validated for cervical and vaginal self‐samples. We used an increased PCR cycle threshold (Ct) of 38.3 for all genotypes to determine positivity which was demonstrated to increase the sensitivity for detecting HPV in first‐void urine by the Predictor 5.1 study [16]. Onclarity is a PCR amplification assay with an internal human β‐globin gene control that detects all 14 high‐risk HPV types: six types are individually genotyped (16, 18, 31, 45, 51, and 52), and the other types are reported in groups (33, 58), (56, 59, 66), and (35, 39, 68). HPV positive samples were further tested using the S5 methylation classifier which combines methylation of both host and viral DNA. It tests for methylation across 3 CpG sites of the host tumour suppressor gene *EPB41L3* and methylation of the viral genes (L1 and L2) of HPV16, HPV18, HPV31, and HPV33 [17]. Residual urine was frozen for future research.

### 2.3 Triage for Those Who Test HPV Positive

There are several options for triaging HPV positive participants [2]. To avoid a speculum‐taken cervical sample for cytology or potential overtreatment of immediate colposcopy in women with transient infection, we chose to repeat the urine HPV test after at least 6 months and refer only those with persistent HPV infection to a colposcopy clinic for further tests and treatment as necessary. Persistent HPV infection is a risk factor for the development of pre‐cancer and a marker of its presence [18].

Given that some women had not been screened for many years and may be harboring disease, a molecular triage test on the initial sample allowed identification of those warranting immediate referral to colposcopy instead of waiting 6 months for retesting. DNA methylation tests have only been used in research studies, and there is limited data on older women [19], where positivity thresholds may differ as methylation has been shown to increase with age [20]. In our study, an average percentage methylation across the 3 CpG sites of *EPB41L3* of 5.5% or more was chosen as the threshold for immediate referral to colposcopy. This novel threshold was chosen based on our unpublished data where over 90% of cancers in a large collection [21] (Nedjai, personal comm) were above this threshold compared with 30% (29/97) of CIN3 cases and only 2.1% (17/800) of the HPV positive controls in the ARTISTIC trial [22] (Gilham, personal comm). We therefore expected very few HPV positives without disease to be referred immediately to colposcopy.

### 2.4 Colposcopy Management and Follow‐Up

Results are returned to participants by letter. Those who have been referred to our study colposcopy clinic are prescribed topical oestrogen for 2–3 months prior to their appointment to make the examination more comfortable and to increase the visibility of the transformation zone [23]. There is limited evidence‐based guidance on how to manage older women with persistent HPV infections [24]. Our panel of colposcopists recommended a cervical sample to be taken for HPV testing and cytology followed by directed biopsies if disease was present or multiple biopsies if there were no obvious lesions and the transformation zone could be visualised. Women with a negative cervical HPV sample and normal colposcopy are discharged, and those positive for cervical HPV with or without cytological or histological abnormalities are managed at colposcopy as per clinical guidelines [25].

### 2.5 Sample Size and Statistical Analysis

In the first phase, 3000 women were invited, giving greater than 80% power at *p* < 0.05 to detect a 10% or greater difference in response rates between screening history groups. The response rate was expected to be higher among those who had been previously well screened [26]. To ensure adequate numbers of those who were previously inadequately screened, the potential participants were stratified by whether they attended for their last screening invitation when aged 60–64. Nationally, around 60% of women currently aged over 65 attended for their last invited cervical screening test at age 60–64 [15]. Our aim was to send 50%–55% of the invitations to those who had not been screened since their 60th birthday. The proportion screened at age 60–64 varied by GP practice, but in most cases, all those who had not been screened since their 60th birthday were invited, plus a random sample of the remainder.

Quintile of area‐level index of deprivation was derived by linking the individual's postcode to area‐level census data for the Income Deprivation Affecting Older People Index [27]. Response rate was the primary outcome measure of interest. Uptake rates were compared by randomisation arm using an intention‐to‐treat (ITT) analysis, regardless of the actual method of reminder. Logistic regression was used to explore characteristics of those responding and to account for confounding: Odds ratios from a fully adjusted model including age group, area‐level deprivation, screening history, and ethnicity were compared to crude odds ratios.

### 2.6 Patient Involvement

A patient group was involved in the study design, reviewing and revising the study materials.

## 3 Results

**FIGURE 2:**
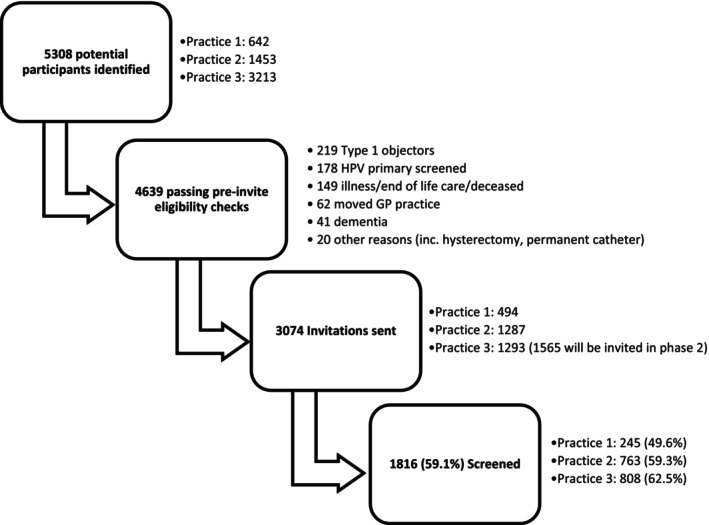
Consort diagram.

A total of 5308 potential participants were identified from three GP practices in Hull and Manchester (Figure 2). All participants from the first two practices were included regardless of screening history. A random sample of those screened since age 60 was excluded from the invitation list of the third practice to ensure that an adequate proportion of under‐screened women were invited (see Sample Size section above).

The medical record search was designed to exclude the majority of ineligible patients (mainly those having had a full hysterectomy), but pre‐invite checks excluded an additional 669 potential participants, mostly due to being type 1 objectors (those who have opted out of their information being used for research purposes), illness, or having been screened previously with HPV primary screening. A total of 4639 participants were deemed eligible, and 3074 invitations were sent as part of the first phase of the project, with 1816 (59.1%) returning a urine sample for HPV testing (Figure 2).

Those from more affluent areas were more likely to accept the invitation, with response rates increasing from 48.3% in the most deprived areas to 64.4% in the most affluent (*p*‐trend < 0.0001). Those who had previously been screened at age 60–64 were also more likely to respond (71.2%) compared to those who did not respond to their last screening invitation from the NHSCSP (39.4%, *p* < 0.0001, Table 1). In the crude analysis, those aged 60–64 were less likely to respond, but their response rate was similar to those aged 65–74 after adjusting for screening history.

**TABLE 1:** Number (%) returning a first‐void urine sample by age, area‐level deprivation and cervical screening history. Crude and adjusted odds ratios from logistic regression models are also shown.

	Number invited (column %)	Number (%) returning a urine sample	Crude odds ratio	Adjusted odds ratio	Adjusted *p* ^a^
*Age*					< 0.0001
60–64	323 (10.5%)	133 (41.2%)	0.53 (0.41–0.69)	1.65 (1.23–2.23)	
65–69	841 (27.4%)	532 (63.3%)	1.31 (1.09–1.59)	1.87 (1.52–2.31)	
70–74	974 (31.7%)	620 (63.7%)	1.34 (1.11–1.61)	1.54 (1.26–1.87)	
75–79	936 (30.4%)	531 (56.7%)	1.0	1.0	
*Area‐level deprivation*					< 0.0001
Q1 (most deprived)	287 (9.4%)	139 (48.3%)	0.52 (0.40–0.67)	0.54 (0.41–0.71)	
Q2	213 (7.0%)	98 (46.0%)	0.47 (0.35–0.63)	0.53 (0.39–0.73)	
Q3	613 (19.9%)	327 (53.3%)	0.63 (0.52–0.77)	0.70 (0.57–0.86)	
Q4	690 (27.6%)	434 (62.9%)	0.94 (0.77–1.14)	1.02 (0.83–1.25)	
Q5 (least deprived)	1260 (41.1%)	811 (64.4%)	1.0	1.0	
*Screening history*					< 0.0001
Last screened age 60–64	1901 (61.8%)	1354 (71.2%)	3.81 (3.27–4.44)	4.13 (3.46–4.94)	
Last screened before age 60 or no record of screening	1173 (38.2%)	462 (39.4%)	1.0	1.0	
*Ethnicity*					0.003
Ethnically diverse/unknown ethnicity	289 (9.4%)	135 (46.7%)	0.58 (0.45–0.73)	0.67 (0.52–0.88)	
White British	2785 (90.6%)	1681 (60.4%)	1.0	1.0	
All invited	3074 (100%)	1816 (59.1%)			

^a^ *p*‐Value for trend given for age and area‐level deprivation. Adjusted model is adjusted for all variables in the table.

Most (90.6%) of those invited were recorded as white British on their GP records, 7.5% had unknown ethnic origin, and just 1.9% were recorded as ethnically diverse. Response was higher among those recorded as white British (60.4% vs. 46.7% among not known/ethnically diverse, *p* = 0.003).

Table 2 shows the response rates to the invitation stratified by the randomly assigned method of follow‐up. The response rate was higher among those randomly allocated to receive a telephone call reminder (61.5%) compared to those randomised to receive a text message (56.7%, OR = 1.22, 95% CI: 1.06–1.41, *p* = 0.007). The largest difference in response was among those who were not previously adequately screened, where the response rate was 35.5% in the text arm and 43.4% in the telephone arm (an increase of 7.9%), compared to a more modest increase among those who had been previously screened at age 60–64 (an increase of 3.3%, 72.9% vs. 69.6%).

**TABLE 2:** Number (%) returning a first‐void urine sample by randomised follow‐up method (intention to treat).

	Telephone	Text message
*Age*		
60–64	76 (46.9%)	57 (35.4%)
65–69	263 (66.4%)	269 (60.4%)
70–74	303 (64.7%)	317 (62.6%)
75–79	283 (59.3%)	248 (54.0%)
*Area‐level deprivation*		
Q1 (most deprived)	75 (52.1%)	64 (44.8%)
Q2	47 (49.5%)	51 (43.2%)
Q3	160 (56.1%)	167 (50.9%)
Q4	206 (62.2%)	228 (63.5%)
Q5 (least deprived)	432 (67.4%)	379 (61.2%)
*Screening history*		
Last screened before age 60	251 (43.4%)	211 (35.5%)
Last screened age 60–64	674 (72.9%)	680 (69.6%)
All invited	925 (61.5%)	891 (56.7%)

Ninety percent of those allocated received a text message reminder as approximately 10% did not have a valid mobile number on their record. Only 49% of those allocated to the telephone arm answered the telephone and were spoken to. We failed to reach 36% of potential participants, and the remaining 15% had already agreed to take part before the telephone call.

Feedback forms were received with 1751 (96.4%) of the samples. Most women (86.3%) found the test quite easy or very easy to do and 96.9% found the instructions quite or very easy to understand. Most women (90.0%) expressed a preference for continued screening into older age, 50.8% with any test and 37.9% with an at‐home test. The most common reasons given for preferring the at‐home test were that it was painless, convenient, easy, private, and did not require them to go out.

## 4 Discussion

### 4.1 Main Findings

We have shown that inviting older women for a catch‐up urine HPV test is well‐received, with 59.1% of those invited returning a urine sample for testing. Those from more affluent areas were more likely to respond to our invitation (61.2% in the most affluent areas falling to 44.8% in the most deprived, *p*‐trend < 0.0001). The strong relationship between deprivation and attendance for screening mirrors national data, where coverage is highest in the most affluent areas at all ages [28]. Women aged under 65 had not responded to their last NHS CSP invitation (by definition), probably explaining their lower response. Response was higher among those followed up with a phone call, with almost 5% absolute difference in response rate (61.5% vs. 56.7%, *p* = 0.007). Most of those who returned a kit gave positive feedback and were pleased to be offered screening. Participants had no difficulty understanding the instructions and found the Colli‐Pee device easy to use. They liked that they could do the test from home, without any embarrassment or pain.

### 4.2 Strengths and Limitations

The first year of Catch‐Up Screen, the largest study of HPV testing in women of post‐screening age in the UK, allowed response rates to be estimated and follow‐up options explored. Its strength lies in sending out over 3000 invitations and screening over 1800 women. Participants were invited from primary care lists, and minimal exclusions were imposed.

Our ITT analysis compared a text and telephone call reminder, but 36% of the telephone arm could not be reached, so we have underestimated the potential effect. Text messages were usually sent out earlier than telephone reminders, reflecting the time required to achieve successful participant response by telephone. A per‐protocol analysis was therefore not done because 15% of participants in the telephone reminder arm had already returned their sample before they could be contacted.

The results may not be generalisable since we invited participants from just three GP practices where the vast majority were white British. One small practice was included where over half of the patients resided in neighbourhoods linked to the most deprived quintile. The other two practices were based in more affluent areas, so a lower response rate may be seen among more diverse populations. A larger proportion of participants from ethnically diverse backgrounds will be included in the second phase of recruitment where women from at least 20 general practices will be invited.

### 4.3 Interpretation

Most screening programmes stop screening women around age 65, resulting in limited data concerning older populations. In Australia, the coverage is 49% among 70–74 year olds [8] which may increase following the recent introduction of self‐sampling. The Danish national catch‐up of those born before 1948 yielded a response rate of 40% among those aged 69–78 attending for a clinician‐taken HPV test [29]. A smaller study inviting 70‐year‐olds in Sweden to attend general practice for screening yielded a 46% response [30]. Our response rates are slightly higher than those reported in these programmes, reflecting the likely benefit of directly mailed at‐home self‐sampling kits over clinician‐taken sampling.

Data among older women regarding barriers to screening is lacking [31] but may be compounded by a lower perception of risk [32]. We designed our information leaflet specifically for older women explaining that screening applies to anyone previously sexually active [10] and highlighting the benefits of HPV testing, even for those adequately screened in the past. In addition, directly mailed self‐sampling kits avoid a speculum examination, which many older women find painful secondary to post‐menopausal reduced oestrogen levels [11], as well as logistical issues booking appointments at their GP surgeries. Our findings are consistent with a randomised trial among cervical screening non‐attenders aged 50–64 years in London showing that self‐sampling and non‐speculum clinician‐taken sampling substantially increase uptake [33].

Urine‐based cervical screening is not currently approved by the NHS CSP, but vaginal self‐sampling will be rolled out for non‐attenders in 2026. The HPValidate project has identified several vaginal swab workflows which give adequate performance for use in the NHS CSP [34]. Urine‐based testing has achieved similar performance to these vaginal swabs and therefore is a viable, less invasive alternative [12, 14]. Furthermore, urine may be preferable to certain populations: A survey among those participating in the YouScreen study of non‐attenders showed that older women and those from ethnically diverse groups were more likely to prefer urine sampling over vaginal self‐sampling [35].

The proportion of women in England aged 50–64 attending for screening within the last 5.5 years has decreased from 80.1% in 2011 to 76.2% in 2019 and 74.3% in 2024 [36]. A national survey found that nearly 40% of non‐attenders aged 50–64 had made the decision not to attend for further screening [37]. An analysis of the pre‐pandemic decline in coverage estimated that each additional Full Time Equivalent (FTE) practice nurse is associated with a 2% absolute increase in coverage [28], suggesting that healthcare cuts may be at least partially responsible for the downward trend. A large Danish population‐based study offered the choice of a clinician‐taken cervical sample or a vaginal self‐sample to women aged 65–69 who did not have an exit test (when aged 60–64) and achieved a 62% response. Overall, 29% of those screened opted for self‐sampling, but this was higher (52%) among those also insufficiently screened in their 50s [38]. In our study, those who had been adequately screened in the past were more likely to respond to our invitation (*p* < 0.0001), but of those who had not responded to their last NHS CSP invitation, 39% returned a urine sample for HPV testing, suggesting that self‐sampling may increase coverage among those attending their last cervical screen.

Personalised reminders have been shown to improve uptake in screening programmes [39] which is consistent with our higher response in those randomised to receive a telephone call. A Scottish study to improve breast cancer screening uptake conducted in 2014 showed the benefit of a telephone call reminder but failed to make contact with 28% of participants [40]. Ten years on, we found that over a third did not answer their phone on three separate occasions, which may reflect hesitancy towards answering calls from unknown numbers. Telephone calls were time‐consuming compared to semi‐automated methods of sending out text messages directly from the GP practice. Despite the increase in response, we did not consider making a large number of telephone calls to be cost‐effective and will focus efforts instead on sending text message reminders to the majority of participants in the second phase of the project.

Recruitment is currently ongoing and at least 18 000 women in total will be invited. This should result in screening 10 000 women, giving the largest population‐based study in the world offering at‐home urine self‐sampling for HPV detection. We estimate that we will detect HPV infections in approximately 500 women who will have colposcopy follow‐up by December 2027. Colposcopy outcomes, including adherence, will provide much needed evidence to inform management guidelines for older women.

## 5 Conclusion

We have offered a catch‐up urine HPV test for cervical screening to women aged 60–79 who had never been screened with an HPV test. Overall, 59% of invited women returned a urine sample for HPV testing. Uptake varied by screening history and area‐level index of deprivation. Among those who declined their last NHS CSP screening invitation, 39% took part in Catch‐Up Screen, suggesting that the offer of at‐home urine‐based self‐sampling has the potential to address decreasing national coverage among older women.

## Author Contributions

C.G. and J.P. conceptualized the study. C.G., J.P., C.R., E.J.D., B.N., and U.M. took part in designing the study. E.J.D., J.C.D., M.F., and J.C. designed the protocol for clinical management and oversaw all clinical aspects of the study. C.R. and C.G. were responsible for all study management. A.Y. and A.M. carried out the data collection. S.V., H.M.‐E., and B.N. conducted the laboratory analysis. C.G. carried out the statistical analyses of the data and wrote the initial draft of the manuscript. All authors critically reviewed and revised the manuscript and approved the final version.

## Funding

Catch‐Up Screen was funded by Yorkshire Cancer Research (reference: RA/2021/R2/123) following external peer review. The funder was not involved in conducting the research or writing the paper.

## Ethics Statement

The study was approved by the London Chelsea Research Ethics Committee (ref 22/LO/0854) and the Confidentiality Advisory Group to give Health Research Authority approval (12 July 2023).

## Consent

The authors have nothing to report.

## Conflicts of Interest

DNA Genotek Inc. provided a number of free Colli‐Pee kits, but played no role in conducting the research, data analysis, or writing the paper. The authors declare no conflicts of interest.

## Data Availability

The data that support the findings of this study will be made available after study completion upon reasonable request to the corresponding author.
